# Core-stability over networks with widespread externalities

**DOI:** 10.1007/s10479-022-04669-5

**Published:** 2022-03-23

**Authors:** László Á. Kóczy

**Affiliations:** 1 Institute of Economics, Centre for Economic and Regional Studies, Tóth Kálmán u. 4., Budapest, 1097 Hungary; 2 Department of Finance, Faculty of Economic and Social Sciences, Budapest University of Technology and Economics, Műegyetem rkp. 3, Budapest, 1111 Hungary

**Keywords:** Externalities, Networks, Recursive core, Social bubble, C71, C72

## Abstract

The Covid-19 epidemic highlighted the significance of externalities: contacts with other people affect the chances of getting infected for our entire network. We study endogenous network formation where not only players or pairs but larger coalitions can, cooperatively change the network. We introduce a model for coalitional network stability for networks with widespread externalities. The network function form generalises the partition function form of cooperative games in allowing the network to be taken into account. The recursive core for network function form games generalises the recursive core for such environments. We present two simple examples to illustrate positive and negative externalities. The first is of a favour network and show that the core is nonempty when players must pay transfers to intermediaries; this simple setting also models economic situations such as airline networks. The second models social contacts during an epidemic and finds social bubbles as the solution.

## Introduction

Networks are used to describe a wide class of social and economic situations. In a social network the nodes are individuals, and the edges or links may be trading possibilities (Corominas-Bosch 2004) channels of information and monetary transfers in an informal insurance setting (Bloch et al. 2008), but we may also think of physical networks, such as road or telecommunication networks (Altman et al. 2006; Roughgarden 2007). Csercsik and Kóczy (2017) describe a network where the edges are power lines connecting power stations and consumers. The task to form balancing groups (where production equals consumption) naturally leads to a cooperative game over the given network. In practice, however, the players do not only use the network, but participate in its maintenance, initiate projects to build new or eliminate old, not needed connections. Our contribution is a new model where the endogeneity of the network is explicitly addressed.

Since Jackson and Wolinsky (1996) we are especially interested in the emergence of stable networks, networks that persist. In this seminal paper stability is driven by the persistence of links: while any player can refuse to maintain a link and thus any player can severe a link, only pairs of players can build new links. This leads to the natural concept of pairwise stability. This concept defines a wide class of stable networks. While these networks are immune to changes by single players or pairs, pairwise stability does not check for possible deviations by larger groups. Dutta and Mutuswami (1997) and Jackson and van den Nouweland (2005) consider strong Nash equilibria (Aumann 1959), where players are permitted to make coordinated, but noncooperative deviations. Such stable networks are very difficult to find, indeed strong Nash equilibria are rather rare and so several alternative models have been proposed. In the noncooperative setting one must very clearly specify the players’ strategies and their abilities to change them. For instance, Calvó-Armengol and İlkılıç (2008) consider the relation of equilibria under single link building and multilink severance. A cooperative approach allows to step over this issue. Ju (2013) considers coalitional cooperation in networks, but with exogenous networks and more limited means of cooperation. Ours is a general, cooperative approach.

Already Jackson and Wolinsky (1996) find seemingly inherent conflict between stability and efficiency: unless we insist on such an extreme allocation rule as the egalitarian there does not, in general, exist a network that is both stable and efficient. There is a conflict between the—selfish—interests of the parties forming the link on the one hand and effect on social welfare, that is, the externalities thus generated to the entire network. Jackson and Wolinsky (1996) assume that there are costs to forming links, but there are widespread benefits of being better connected. Morrill (2010) assumes that a new relationship is beneficial to the parties, but that the new connection may harm the rest of the network. The case of an employment network is cited, where acquaintances help players to new jobs, but the value of a connection depends on its exclusivity; the co-author network of Jackson and Wolinsky (1996) is another perfect example. Möhlmeier et al. (2016) combine the connection model with the co-author model allowing for positive externalities of link formation via the connections it provides, while increasing the congestion at the end nodes creating negative externalities at the neighbours. Buechel and Hellmann (2012) show that positive externalities may lead to under-connected, while negative externalities to over-connected stable networks (with both terms formally defined). We take a different, purely cooperative approach, where coalitional improvements are possible: a coalition can freely rearrange its internal structure of connections and may also jointly optimise the connections to other players. As a special case, deviations by the grand coalition correspond to Pareto-improvements therefore stability implies efficiency. This is different from a number of models where focus is on the allocation of the benefits of a given network (Park and Ju 2016).

While coalitional deviations are also accounted for under strong stability, there is a fundamental difference between cooperative and noncooperative deviations. Under strong stability, coalitional deviations are unilateral and simultaneous. Our cooperative approach is based on the core. The core is a static concept: when a core allocation is proposed, it is accepted by all without protest. On the other hand, deviations from a non-core proposal are modelled in a dynamic way. If the proposal on the table does not meet the expectations or demands of one of the coalitions, it makes a threat of leaving the full cooperation if its demands cannot be met. If the threat works, the original proposal is abandoned, and a new one is made. If the threat does not work, the coalition in question leaves the joint agreement and begins to act according to its own interests. Due to the externalities its value will depend on the network the remaining players form.

The structure of the paper is, accordingly, the following. First we introduce the general notation, introduce the game form we use to model the coalitional network games and recall the recursive core that inspires the solution concept that is introduced in the next section. We study two simple networks at length, determine the stable structures and discuss their possible applications.

**Fig. 1:**
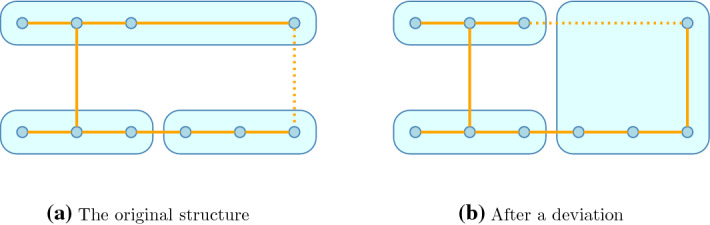
Clusters with their cooperation network—dotted links are discontinued for economic reasons

## Preliminaries

### A motivating example

We consider an industry with research clusters (Fig. 1a): groups of firms with a well-established legal framework to facilitate knowledge transfer. There is also a network of cooperation among firms. There is, however, a substantial difference between a link that runs within the cluster and one that crosses cluster boundaries: the cluster organisation facilitates cooperation within but external cooperation has substantial additional costs. We do not specify a utility function but assume that cooperation has decaying spillovers over the network. Then the dotted link between the last members of the Top and Right clusters benefits primarily those near and overall the top cluster may find the costs higher than the benefits—the benefits in the Right cluster are, of course, ignored here.

Since the members of the Top cluster make joint decisions, they do not approve the dotted link on the right. This link is severed. This, however, does not change the interests of the Right cluster: they still want to form the link. If, for whatever reason, the Top cluster changed its mind, the link could be formed without any action from right’s part.

Of course, the high legal costs of the dotted link could be removed by making it internal. Figure 1b shows the top-right firm joining the Right cluster and the (direct) link to the rest of the Top cluster is discontinued.

### The outline of the model

We consider a cooperative game of network management. Players can, cooperatively, build or cancel links, and the coalitional payoffs depend explicitly on the graph, as well as the coalition structure. Our notion of coalition stability is based on the idea of the (coalition structure) core. When some coalitions deviate from the current structure they can (i) arbitrarily restructure internal links, (ii) severe any outgoing links (iii) propose new outgoing links and (iv) allocate the value of the coalition among its members. The value of the coalition can only be determined once the entire graph is known so we determine the structure of the remaining, residual players.

There are several possible approaches to take. By considering strong Nash equilibria (Dutta and Mutuswami 1997) essentially assume that the structure remains unchanged. This is, however, not very likely given the widespread externalities present in the network: the deviation will change the payoff of the remaining players, who will react to the externalities in a complex way: they might form coalitions who also optimise their internal structure as well as choose the outgoing links they want to have or keep. The value of the deviating coalition will, in turn, also depend on the reaction chosen by the residual players so it is natural to take a conservative approach and expect the worst: if the the deviation is profitable in the worst case, it is surely profitable.

While this approach is very conservative from the viewpoint of the deviating players, the resulting set of coalitionally stable networks may include networks that only appear stable because of this assumption of extreme pessimism. In other words, extreme pessimism corresponds to extreme optimism regarding stability. More importantly, this approach ignores the interests of the residual players. So, along the lines of the recursive core (Kóczy 2007) we assume that the residual players play a similar game and pessimism is applied only to the resulting, often unique coalitionally stable network or rather the payoffs induced by these residual structures.

We do not assume that the deviating coalition breaks all ties with the rest. The residual problem may therefore contain some outgoing links: these links can be broken, but no new outgoing links can be built. The latter follows from the fact that this would require mutual consent, that is, endpoints belonging to the same coalition. The outgoing links will be formally connecting to an artificial, non-strategic player 0 representing the outside world; when a set of players leaves the game, the links to this set are simply remapped to end at 0. A player may have links to more than one of the deviating players and these links may have very different implications therefore we keep all of these. As a result there may be multiple links to the outside world requiring us to use somewhat more general notation. This more general notation, on the other hand, permits us to consider rather general problems. For instance, in the case of a power network the different parallel arcs may be power lines with different transmission capacities and solving this game may determine not only where the lines should be built, but also what their capacities should be. In the case of an existing line an upgrade could be modelled likewise.

Now we move on to the formal model of this graph.

### Formal model

We consider a universe *U* of players. Let $N=\left\{ 1,2,\dots ,n\right\}$ denote a finite carrier, $2^N$ the set of all subsets, called coalitions. For a set *S* let ${\overline{S}}$ denote its complement $N{\setminus } S$. Let $\Pi$ denote the set of partitions of *N* into non-overlapping subsets, partitions of $S\subseteq N$ are denoted $\Pi (S)$. An embedded coalition is a pair $(C,{\mathcal {P}})$ such that *C* is a coalition, ${\mathcal {P}}$ is a partition and $C\in {\mathcal {P}}$. The set of embedded coalitions is denoted by ${\mathcal {E}}$.

**Fig. 2:**
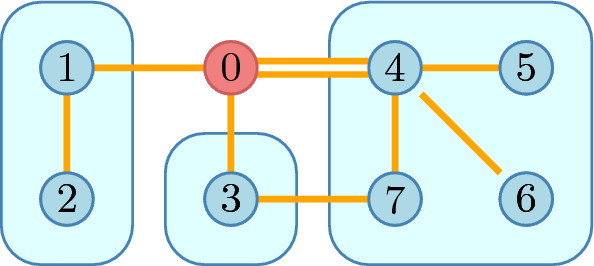
An example structure with the outside player 0

We consider *open networks*, networks with an additional player 0 representing the “outside world.” It will be useful for our model to allow a player *i* to have multiple links to 0 and therefore we consider a *multigraph* over $N_0=N\cup \left\{ 0\right\}$. While a multigraph could be more general, we assume that any player *i* has at most a single link, denoted ${\overline{ij}}$, to each $j\in N{\setminus } \left\{ i\right\}$ and possibly multiple links to 0 (Fig. 2). The latter links can be of two types:

- a link $\overrightarrow{ij}$ expressing a unilateral interest in building the link ${\overline{ij}}$ and
- links to nodes beyond the current player set *N*.

With this we acknowledge that the network may reach beyond *N* and we may label these edges such as $\overrightarrow{ik}$ where $k\in U{\setminus } N$ is a non-strategic neighbour.

We denote the set of possible links by $L=\left\{ {\overline{ij}}, \overrightarrow{ij}, \overrightarrow{ji}\mid i,j\in N\right\} \cup \left\{ \overrightarrow{ik}\mid i\in N, k\in U{\setminus } N\right\}$. Then $2^L$ is the set of possible networks; a typical network is denoted by $\ell$. Notice that the link $\overrightarrow{ij}$ for $i,j\in N$ really connects *i* and 0 but of all the links connecting these nodes, $\overrightarrow{ij}$ is the intention to connect to *j*.

We say that $\pi = \left\{ \overline{i_{k-1}i_k}\right\} _{k=1}^m$, where $i_k\in N$ is a path of length *m* and the distance between nodes *i* and *j*, denoted *s*(*i*, *j*) is simply the length *m* of the shortest path with $i=i_0$ and $j=i_m$. Notice that paths must connect players via players, 0 is not permitted here.

**Fig. 3:**
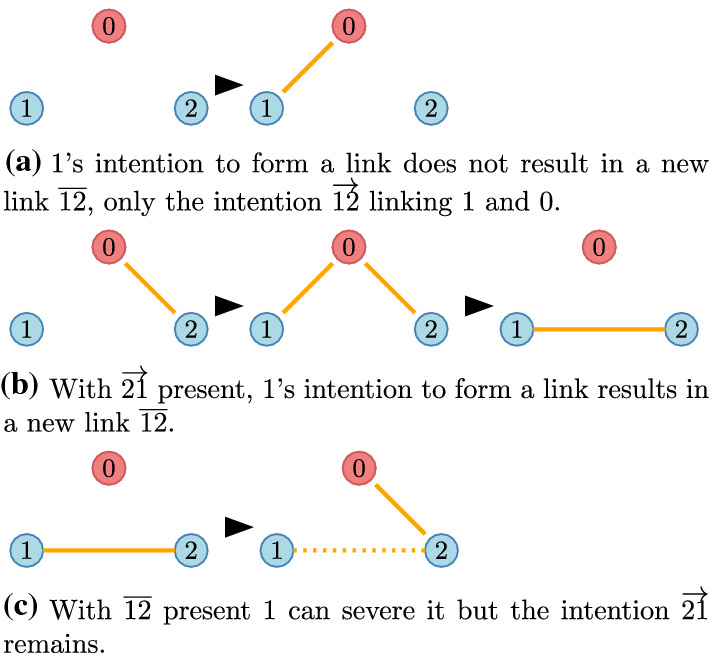
Available actions for player 1

We assume that the network is endogenous: players can build and destroy links (Fig. 3). Following the mainstream network formation literature, we assume that a player can severe any of the links connecting to a neighbour but can only create a link to a neighbour if she agrees.

- A new intention $\overrightarrow{12}$ to build a link can be expressed but results in no link ${\overline{12}}$ unless the intention $\overrightarrow{21}$ already present (Fig. 3a).
- When $\overrightarrow{12}$ and $\overrightarrow{21}$ are both present, these are replaced by the link ${\overline{12}}$ (Fig. 3b).
- An existing link ${\overline{12}}$ can be severed (Fig. 3c). In this case the other player’s intention is modelled by a remaining link $\overrightarrow{21}$ to the “outside world” 0.

**Fig. 4:**
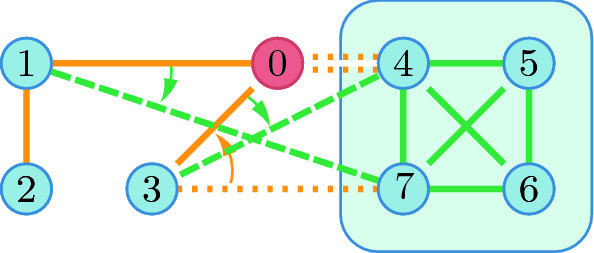
Available actions for a coalition: freely changing internal links (green), severing outside links (dotted orange), and turning external intentions into links (dotted green: $\overrightarrow{34}$ into ${\overline{34}}$ and $\overrightarrow{17}$ into ${\overline{17}}$). (Color figure online)

Coalitions can build or cancel any of the internal links among its members, destroy or propose outgoing links (Fig. 4). A proposed will only materialise if the recipient also intends to build that link, while severed existing links are reduced to an intention on the other side.

We define the value of a coalition of players in a network by the network function

2.1 $$\begin{aligned} V: {\mathcal {E}}\times 2^L\longrightarrow \mathbb {R}. \end{aligned}$$

This function assigns a real value to each coalition in a network, a value $V(C,{\mathcal {P}},\ell )$ to each coalition *C* embedded in partition ${\mathcal {P}}$ given a network $\ell \subseteq L$.

The triple (*N*, *L*, *V*) is a cooperative game in *network form* or simply a game.

For the vectors *a*, *b* and coalition $C\subseteq N$, let $a_i$ denote the coordinate of $i\in N$, $a_{-i}$ the vector *a* with the *i*th component removed, $(b_i,a_{-i})$ the vector *a* with the *i*th component replaced by that of *b*; $a_C=(a_i)_{i\in C}$ the restriction of *x* to *C*; finally $a>b$ if $a_i\ge b_i$ for all $i\in C$ and $a\ne b$.

We call a triple $\omega =(x,{\mathcal {P}},\ell )$ consisting of a payoff vector *x*, a partition ${\mathcal {P}}$ and $\ell \in L$ an *outcome* if it satisfies the following:

$$\begin{aligned} \sum _{i\in C} x_i=V(C,{\mathcal {P}},\ell ),\quad \text {for all}\quad C\in {\mathcal {P}}, \end{aligned}$$

in other words, transfers are permitted within coalitions.

Let us denote the set of outcomes in (*N*, *L*, *V*) by $\Omega (N,L,V)$. The aim of this paper is to find the outcomes that cannot be improved upon by any coalition of players including the grand coalition.

### The recursive core

Before we move to the definition of the core for games in network form, first recall the recursive core for partition function form games.

In a partition function form game (see Kóczy 2018, for a recent survey) whether a coalition benefits from deviating depends on the induced partition of the players. In the projective core, no reaction is assumed (Abe and Funaki 2020). The $\alpha$-core (Aumann and Peleg 1960) assumes that a coalition deviates only if it gets a higher payoff irrespective of the induced partition, so the deviation must be profitable even under the most adverse conditions. The core stability (Shenoy 1979) is more permissive: a coalition deviates if any of the induced partitions gives a higher payoff. In the $\gamma$-core (Chander and Tulkens 1997) the coalition must face individually best responses. Here we recall the concept of the *recursive core* (Kóczy 2007, 2009), that allows the remaining, residual players to freely react and form a core-stable partition before the payoff of the deviating coalition is evaluated.

First we define the *residual game* over the set $R\subsetneq N$. Assume ${\overline{R}}=N{\setminus } R$ have formed ${\overline{{\mathcal {R}}}}\in \Pi ({\overline{R}})$. Then the residual game $(R,V_{{\overline{{\mathcal {R}}}}})$ is the partition function form game over the player set *R* with the partition function given by $V_{{\overline{{\mathcal {R}}}}}(C,{\mathcal {R}})=V(C,{\mathcal {R}}\cup {\overline{{\mathcal {R}}}})$.

#### Definition 1

(*Recursive core* Kóczy 2007) For a single-player game the recursive core is trivially defined. Now assume that the recursive core *C*(*N*, *V*) has been defined for all games with $\left| N\right| <k$ players. We call a pair $\omega =(x,{\mathcal {P}})$ consisting of a payoff vector and a partition ${\mathcal {P}}\in \Pi (N)$ an outcome. Let us denote the the set of outcomes in (*N*, *V*) by $\Omega (N,V)$. Then for an $\left| N\right|$-player game an outcome $(x,{\mathcal {P}})$ is dominated if there exists a coalition *Q* forming partition ${\mathcal {Q}}$ and an outcome $(y,{\mathcal {Q}}\cup {\overline{{\mathcal {Q}}}})\in \Omega (N,V)$, such that $y_Q> x_Q$ and if $C({\overline{Q}},V_{{\mathcal {Q}}})\ne \varnothing$ then $(y_{{\overline{Q}}},{\overline{{\mathcal {Q}}}})\in C({\overline{Q}},V_{{\mathcal {Q}}})$. The *recursive core* *C*(*N*, *V*) of (*N*, *V*) is the set of undominated outcomes.

The recursive core is well-defined, though it may be empty.

## The recursive core for games in network function form

Our new coalitional stability concept is introduced in this section. The main lines of this concept are similar to those of the recursive core except that the payoff of players or coalitions is also dependent on the (endogenous) network players form, but the more general game requires some more complex notation and terminology. First we present this notation, as before, we define the way to derive a residual network game and finally present the recursive core for network games.

**Fig. 5:**
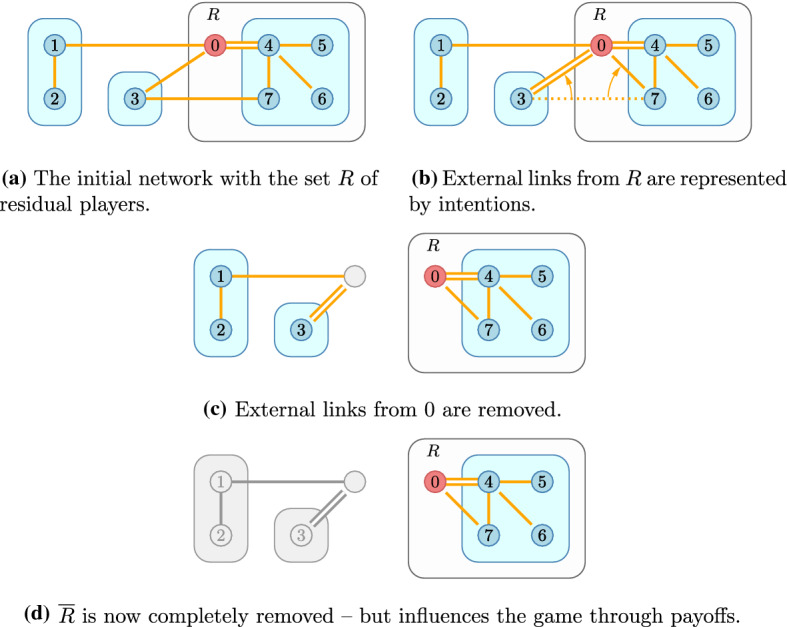
Establishing the residual network for *R*

### Residual games

Now assume that a coalition ${\overline{R}}$ has left the game forming partition ${\overline{{\mathcal {R}}}}$ and network $\ell _{{\overline{R}}}\subseteq L_{{\overline{R}}}$, where $L_{{\overline{R}}}=\left\{ {\overline{ij}}\left| {\overline{ij}}\in L, \left\{ i,j\right\} \cap {\overline{R}}\ne \emptyset \right. \right\}$ is the restriction of the set of feasible links to ${\overline{R}}$. Recall that $\ell _{{\overline{R}}}$ contains arcs within ${\overline{R}}\cup \left\{ 0\right\}$, but also arcs that lead to the remaining players in *R*. Depending on the choice of players in *R* some of these existing, or proposed will be abandoned, some may be accepted or maintained. Likewise the consequence of having an outside link from *R* will depend on the interest at the other end in maintaining this connection. Since this interest is already known, knowing $\ell _{{\overline{R}}}$ it is possible to calculate the effect simply by knowing the choices in *R*. Indeed, given these decisions the problem the remaining players in *R* face is similar to the original game: it is also a game in network function form, just a little smaller one. In the following we define this *residual* game.

The residual game is a triple $(R,L_R,V_R)=\left( R,L_R,V_R^{\ell _{{\overline{R}}},{\overline{{\mathcal {R}}}}}\right)$ where $V_R^{\ell _{{\overline{R}}},{\overline{{\mathcal {R}}}}}$ is the network function of this smaller game. While this is a network function on its own, we derive it from the original network function by re-connecting the residual game with the players who have already quit, with their partition and network.

Using our example network (Fig. 2), we show how the original network is restricted to the residual game in Fig. 5. First we identify the set *R* of residual players. Internal links in $N_0$ are preserved. Links to external players are now pointing to the outside world and are therefore reduced to intentions—on both sides.

Payoffs are the same as in the original game, and to determine these, we must recombine the residual configuration with that of ${\overline{{\mathcal {R}}}}$. Players and partitions are combined as the union of players and partitions, respectively; when the networks are combined matching intentions are remerged.

3.1 $$\begin{aligned} (\ell _{R},\ell _{{\overline{R}}})=(\ell _{{\overline{R}}}\cup \ell _{{R}}){\setminus } \left\{ \overrightarrow{ij}\mid \overrightarrow{ji}\in \ell _{{\overline{R}}}\cup \ell _{{R}}\right\} \cup \left\{ {\overline{ij}}\mid \overrightarrow{ij},\overrightarrow{ji}\in \ell _{{\overline{R}}}\cup \ell _{{R}}\right\} . \end{aligned}$$

Then we can define the network function for the residual game

3.2 $$\begin{aligned} V_R^{\ell _{{\overline{R}}},{\overline{{\mathcal {R}}}}}(C,{\mathcal {P}}_R,\ell _R)=V(C,{\mathcal {P}}_R\cup {\overline{{\mathcal {R}}}},(\ell _R,\ell _{{\overline{R}}})). \end{aligned}$$

### The recursive core

Now we can define the recursive core for network function form games. The definition is analogous to that for partition function form games and is therefore recursive. First the core is defined for a trivial, single player game. Assuming the definition for all, at most $k-1$ player games, we extend the definition to *k* player games.

#### Definition 2

(*Recursive core for network games*) For a single-player game the *recursive core* is trivially defined.

Now assume that the recursive core *C*(*N*, *L*, *V*) has been defined for all games with $\left| N\right| <k$ players and consider an $\left| N\right|$-player game (*N*, *L*, *V*).

We say that the outcome $(x,{\mathcal {P}},\ell )$ is *dominated* if there exists a coalition *Q* forming partition ${\mathcal {Q}}$ with network $\ell _Q$ and $y_Q$ such that *for all* outcomes $\left( (y_Q,y_{{\overline{Q}}}),{\mathcal {Q}}\cup {\overline{{\mathcal {Q}}}},(\ell _Q,\ell _{{\overline{Q}}})\right) \in \Omega (N,L,V)$ satisfying

- $(y_{{\overline{Q}}},{\overline{{\mathcal {Q}}}},\ell _{{\overline{Q}}})\in C\left( {\overline{Q}},L_{{\overline{Q}}}^{\ell _Q},V_{{\overline{Q}}}^{\ell _Q,{\mathcal {Q}}}\right)$ if this core is nonempty, or
- $$(y_{{\overline{Q}}},{\overline{{\mathcal {Q}}}},\ell _{{\overline{Q}}})\in \Omega \left( {\overline{Q}},L_{{\overline{Q}}}^{\ell _Q},V_{{\overline{Q}}}^{\ell _Q,{\mathcal {Q}}}\right) $$ otherwise

we have $y_Q> x_Q$. The *recursive core* *C*(*N*, *L*, *V*) of (*N*, *L*, *V*) is the set of undominated outcomes.

In the terminology of Kóczy (2007), this is the pessimistic version, where pessimism is on the part of the deviating players. There is a corresponding version with optimistic players, where it is sufficient if the deviation is profitable for *any* (thus not all) of the residual (core) outcomes. It is easy to verify that the optimistic recursive core is weakly contained in the pessimistic recursive core.

Note that the elements of the core are always Pareto efficient: Pareto inefficient outcomes are dominated via a deviation of some partition of *N*.

**Fig. 6:**
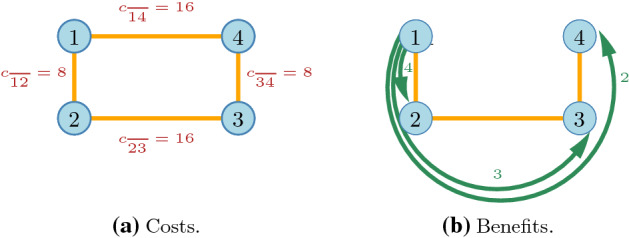
The costs and benefits in Example 1

### Relation to other network formation models

In a seminal paper on network formation Jackson and Wolinsky (1996) introduced *pairwise stability*. A network is pairwise stable if (1) no unconnected pair benefits from linking up and (2) no player benefits from deleting an existing connection.

Our model differs in at least two aspects from pairwise stability. On the one hand, pairwise stability focuses on pairs, while we consider larger coalition, too. This would make our deviation concept more general and the set of stable configurations smaller. Pairwise stability, on the other hand, is a fundamentally noncooperative equilibrium concept as a slightly modified version is presented by Bloch and Jackson (2007) makes it even clearer. In such games there is no discussion about possible reactions as the decisions are made simultaneously. In our model players are more farsighted calculating with the reaction of other players and these reactions influence the profitability of deviations. This aspect makes the two sets of solutions or equilibria mutually non-inclusive in general and applies to most of the network models (Bloch and Jackson 2006), although for special classes of games one or the other may be more permissive.

In order to illustrate the differences, we present the following simple example with 4 nodes.

#### Example 1

In a *connections game* players benefit from being connected to each other. A connection may be direct or indirect, but indirect connections are less valuable. It is costly to be connected and the cost of the connection depends on the distance. We consider a game with four nodes, located at the corners of an elongated rectangle where connections are only possible on the sides. The short connections cost 8, long connections cost 16. The benefit of a direct connection is 4, an indirect one with a single stop is 3 and with two stops is 2 (Fig. 6).

**Fig. 7:**
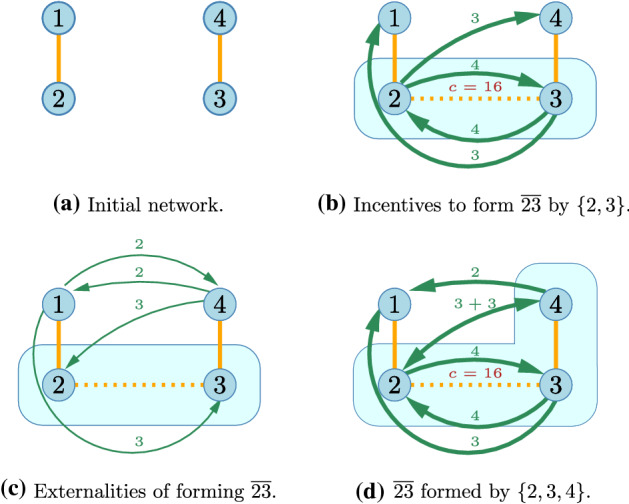
Incentives to form the link ${\overline{23}}$

Immediately note that an empty network is pairwise stable, since the cost of a new link (at least 8) is not less than the benefits (4 for each of the connected nodes). If we would consider this to be a game of network formation, this would be the end of the story.

Suppose now that the two pairs of close nodes are connected and are considering to link up (Fig 7a). Would this connection be built? The benefit of the two players involved is a direct and a 1-stop connection, valued 7, while at least one of them would have to contribute at least 8 to establish the connection so the link is not profitable (Fig 7b). They, however, do not take the positive externalities generated into account (Fig 7c). With coalitions we can bring all the benefited parties on board. It is already sufficient to consider a trio (Fig 7d). The fourth player will keep its connection and the coalition has 5 direct, 3 one-stop and 1 two-stop connections and must pay a long, and 1.5 short connections, so the payoff is 3 exceeding the *ex ante* payoff of 0.

**Fig. 8:**
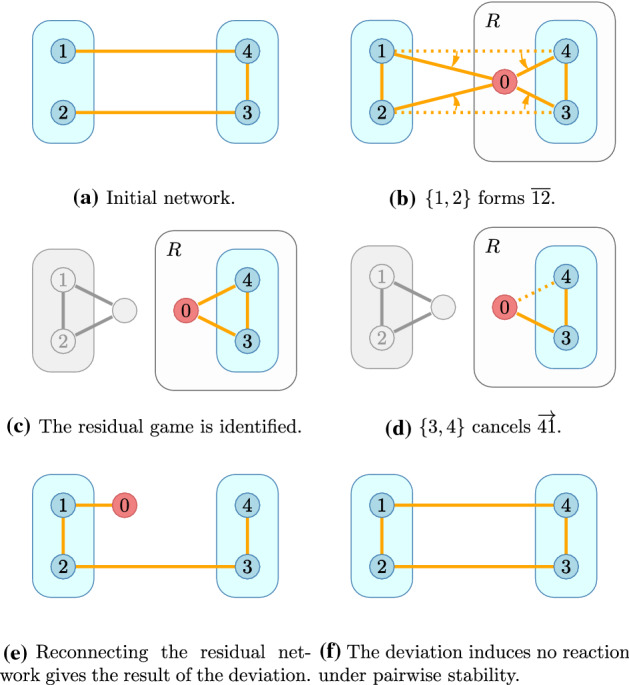
The story of a deviation to form the link ${\overline{12}}$

#### Example 2

Now consider the same game but with a different *status quo* (Fig. 8a). Let ${\mathcal {P}}=\left\{ \left\{ 1,2\right\} ,\left\{ 3,4\right\} \right\}$ and $\ell =\left\{ {\overline{13}},{\overline{24}},{\overline{34}}\right\}$. Should the coalition $\left\{ 1,2\right\}$ build the link ${\overline{12}}$ or not? Initially, the payoff of this coalition is 2. When it deviates (Fig. 8b), a residual game forms (Fig. 8c) and the residual players respond as a coalition (this is a weakly dominant strategy in this game); they can break arcs $\overrightarrow{31}$, $\overrightarrow{42}$ and ${\overline{34}}$. Since they keep ${\overline{34}}$, they can break no, one or both of the long arcs.

The residual coalition prefers to keep only 1 long arc (Fig. 8d). In this (once again symmetric) case both coalitions have 3 direct, 2 one-stop and 1 two-stop connections and pay for 1 short and a half long arc, giving a payoff of $12+6+2-16=4>2$, so the coalition will build the arc in question.

Note that the coalition $\left\{ 1,2\right\}$ could have alternatively severed one of the long arcs themselves but the threat that the residual coalition removes the other long arc (giving a payoff 0) is still there.

Under *pairwise stability* the rest of the network does not react, which corresponds to the case when no arcs are broken (Fig. 8f). In this case both the deviating and the residual coalition has 4 direct and 2 one-stop connections and pays for a sort and two half long arcs, giving a payoff of $16+6-24=-2$. Under pairwise stability the link ${\overline{12}}$ would not be built.

**Fig. 9:**
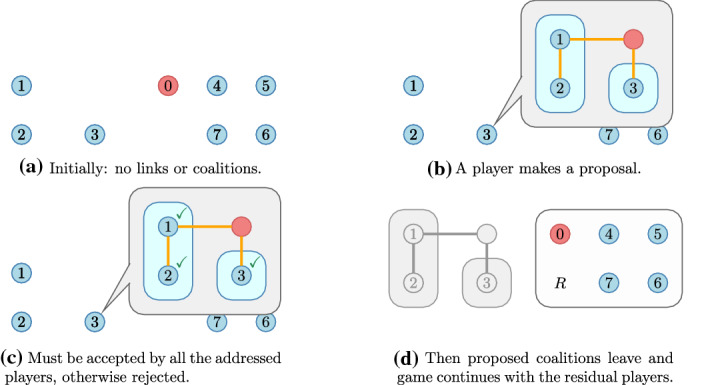
An outline of the coalition and network formation game

## Implementation results

In the following we introduce a noncooperative network and coalition formation game that produces a set of outcomes that are always contained in the recursive core of the network formation game.

### Outline

First, we present an informal outline of the game.

Initially all players are active. Players can make proposals in continuous time. A proposal addresses a subset of the players and specifies their partition, the *shares* each player will get from the coalitions’ payoffs and the network, both internal and outgoing. If each addressed player accepts the proposal, then they leave the game and implement the proposal. Otherwise another proposal is made and this automatically cancels the previous one. The game ends if all players have left, but it may or may not end. Players also get payoffs in this latter case (Fig. 9).

The focus is on stationary strategies and we will look at subgame-consistent strategies (Kóczy 2015), where subgame perfectness is only required around the equilibrium path, while irrelevant subgames are ignored.

### Strategies and states

Consider a game (*N*, *V*) with a player set *N* and a network function *V*. Time *t* is continuous, players can act at any time, but we assume that there is always an open time interval between two actions allowing players to intervene if necessary—this explicitly rules out two actions happening at the same time.

During the game players make proposals to form coalitions. A proposal automatically cancels the previous proposal. When a proposal is accepted by all the players in the proposed coalition, the coalition leaves the game and its members become inactive. Initially all players are active, but let us consider a more general, later stage, when some players have already left: At time *t* let $Q^t\subseteq N$, ${\mathcal {Q}}^t$ and $\ell _Q^t$ respectively denote the set of quitted players by time *t*, the partition and network they have formed—including some intentions to form links to players in $\overline{Q^t}$. In such a case a player *i* can make proposal that specifies both the coalitions, the network and the payoff allocation, formally a probability distribution to allocate coalitional payoffs within the coalitions. Formally a proposal is

4.1 $$\begin{aligned} p^t_i=\left\{ ({\mathcal {P}}^t,\ell ^t,w^t)\left| \begin{array}{l} {\mathcal {P}}^t\in \Pi (P^t), P^t\subseteq \overline{Q^t}, P^t\ni i,\\ \ell ^t\in L_{P^t}, \\ w^t\in \mathbb {R}^{P^t}, w^t\ge 0, \forall P^t_k\in {\mathcal {P}}^t \sum _{j\in P^t_k}w^t_j=1 \end{array}\right. \right\} . \end{aligned}$$

The current proposer is $i^t$ making the proposal $p^t=({\mathcal {P}}^t,\ell ^t,w^t)$ to the players in $P^t$; one that is already accepted by the players in $A^t\subseteq P^t$ (we assume $i^t\in A^t$). At *t* a player *i* can

1. accept the current proposal $p^t$ if $i\in P^t$ and $A^t=A^{t-\varepsilon }\cup \left\{ i\right\}$, where $\varepsilon >0$ is small,
2. make a new proposal, or
3. do nothing.

The strategy $\sigma _i$ of a player *i* specifies a complete protocol of actions for all times and contingencies during the game and $\sigma ^t_i$ the action at *t*. Let $\sigma$ denote the strategy profile collecting the strategies of all players.

*History* *h* is a complete record of (irreversible) events, represented by the sequence $\left\{ \left( {\mathcal {Q}}^{t_k}, \ell ^{t_k}_{Q}\right) \right\} _k$ of states $s^t=({\mathcal {Q}}^t, \ell ^t_{Q},i^t, p^t, A^t)$ at times $t=t_k$ where the partition ${\mathcal {Q}}^t$ has changed. The name is somewhat confusing in the sense that a history covers the past *and* the future.

We assume that $s^0=(\varnothing ,\varnothing ,\varnothing ,\varnothing ,\varnothing )$. How does the game transit between states? Starting from $s^t=({\mathcal {Q}}^t, \ell ^t_{Q},i^t, p^t, A^t)$,

- if $i^t$ makes a new proposal $p^t$, the state becomes $s^t=({\mathcal {Q}}^t, \ell ^t_{Q},i^t, p^t, \varnothing )$;
- when a player *i* (in $P^t$) accepts a proposal, the state becomes $s^t=({\mathcal {Q}}^t, \ell ^t_{Q},i^t, p^t, A^t\cup \left\{ i\right\} )$;
- if all players accept the proposal $p^t=({\mathcal {P}}^t,\ell ^t,w^t)$, the new state becomes $({\mathcal {Q}}^{t}\cup {\mathcal {P}}^t, \ell ^t_{Q}\cup \ell ^t,\varnothing , \varnothing , \varnothing )$;
- finally the game ends if ${\mathcal {Q}}^t\in {\mathcal {P}}(N)$.

Subgames are identified with history truncations $h^t$ to time in $\left[ t,\infty \right)$; let $\sigma \vert _{h^t}$ denote the restriction of strategy $\sigma$ to the subgame $h^t$. Also, if $s=({\mathcal {Q}}, \ell , i, p, A)$, we write ${\mathcal {Q}}(s)$ for ${\mathcal {Q}}$, $\ell (s)$ for $\ell$, etc.

### Payoffs

Only players who leave the game may obtain payoffs; their payoff is determined conservatively, for each coalition, in case some players remain in the game for ever:

4.2 $$\begin{aligned} w_i^tV(Q_k,{\mathcal {Q}},\ell _Q)={\left\{ \begin{array}{ll} w_i^t\min \left\{ V(Q_k,{\mathcal {P}},\ell )\left| {\mathcal {P}}\supset {\mathcal {Q}}, \ell \supset \ell _Q\right. \right\} &{}Q_k\in {\mathcal {Q}}\\ 0&{}\text {otherwise.} \end{array}\right. } \end{aligned}$$

We denote the payoff *i* obtains given a strategy profile $\sigma$ by $x_i(\sigma )$ and by $x_i(\sigma ,s)$ if the game continues from some—off-equilibrium—state *s*. Occasionally, we will want to stress that this is the payoff obtained if no deviations take place and call this the continuation payoff.

### Alternative histories

In a cooperative game, stability is taken care of by the threat of a residual response that makes a deviation non-profitable. If the residual core supports multiple partitions and network configurations this response is nontrivial, and it is not always preferred by all residual players. Still, the pessimism of the deviating coalitions makes them focus on these reactions.

In the noncooperative game the situation is a little different, especially if we focus on stationary strategies. Players playing stationary strategies cannot condition their actions on past events of the game, they, effectively, do not remember what happened. Instead, the current state is explained by an alternative history and players act on this as if it were the real one. The alternative history may or may not be the true history but it is accepted by all players explains the current state of the game. It is not strategically selected in a possibilistic rather than probabilistic way. By acting on this alternative history active players will occasionally get the reaction right and successfully punish deviators.

**Fig. 10:**
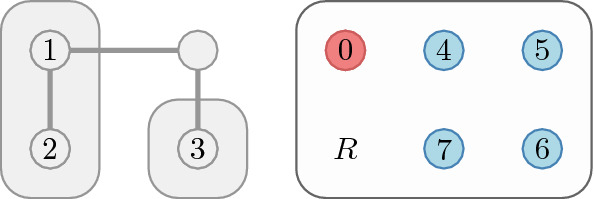
Off equilibrium state with multiple coalitions

#### Example 3

Consider the configuration in Fig. 10 and assume that in equilibrium the entire configuration should form instantly. There are two deviating coalitions, $\left\{ 1,2\right\}$ and $\left\{ 3\right\}$. There are three things that may have happened:

1. The partition $\left\{ \left\{ 1,2\right\} ,\left\{ 3\right\} \right\}$ and the corresponding network was proposed with some weights and this proposal was accepted by all three players. By the assumption that the core is not empty, this deviation is not profitable in the cooperative game and therefore there exists a some *punishment* configuration, let us call this $B_1=B(\left\{ \left\{ 1,2\right\} ,\left\{ 3\right\} \right\} )$ specifying a network and a partition of the remaining players that ensures that the deviating players’ payoffs will be lower than playing their equilibrium strategies. Let $\beta _1$ denote the strategy that results in the configuration $B_1$—for the moment accept that such a strategy exists.
2. It is also possible that first $\left\{ 1,2\right\}$ formed and instead of forming $B(\left\{ \left\{ 1,2\right\} \right\} )$, coalition $\left\{ 3\right\}$ left. In such a situation it is $\left\{ 3\right\}$ that must be sanctioned, by some strategy $\beta _2$.
3. As a last possibility, perhaps $\left\{ 3\right\}$ left first and then instead of applying $B(\left\{ \left\{ 3\right\} \right\} )$, coalition $\left\{ 1,2\right\}$ left. Again, this is the coalition that must be sanctioned, using strategy $\beta _3$.

These $\beta$’s are not necessarily distinct.

There may or may not be a $\beta$ that is universally effective. Notice that in the latter two cases the deviations are not from the equilibrium configuration, but from $B(\left\{ \left\{ 1,2\right\} \right\} )$ and $B(\left\{ \left\{ 3\right\} \right\} )$, respectively. They may represent very different payoff levels.

Instead of trying to find a single punishment, we assume that the remaining players make up a possible history, one that is compatible with either of the above stories. When this is chosen, the corresponding punishment strategy is employed. The possibility of getting the history right keeps players from deviating.

As an important assumption of our model, we assume that players do not keep track of the events of the game. Instead, they look at the current state *s*: if this is one of that states that should emerge under equilibrium gameplay, they continue playing the equilibrium strategies. If, on the other hand, *s* is not such a state, they make up a theory that explains *s*: a made-up history, that is compatible with *s*. Formally, we say that given the current state *s*, players make up one of the possible histories $h(s)\in {\mathcal {H}}(s)=\left\{ h\left| \exists t: h^t(s)=\left( {\mathcal {Q}}(s),\ell (s)\right) \right. \right\}$ compatible with the current state *s*. As the game is played further and the partition ${\mathcal {Q}}^t$ changes, for an off-equilibrium $s'$, the current history is abandoned and another one is made up. This new history may or may not contain *s* at all. Let ${\mathcal {H}}(\sigma ,s)\subseteq {\mathcal {H}}(s)$ denote the histories that are *compatible with* $\sigma$ *and* pass through *s*. Our idea of compatibility is analogous to the intuitive criterion for perfect Bayesian equilibria: In general, we assume that players follow $\sigma$ and only deviate if this makes sense, that is, if the deviation carries the possibility of a payoff higher than the continuation payoff. Assuming rational players, *s* is either a state that is off the map when playing $\sigma$—in which case our argument does not help restricting strategies—or it can be reached by a sequence of equilibrium actions interspersed by deviations with the potential to make a profit. Then the question is whether the subsequent moves prescribed by $\sigma$ are able to turn these hopes for profit down.

Individual payoffs are also affected by the alternative histories. Let $x(\sigma , h)\in \mathbb {R}^N$ denote the vector of payoffs in case $\sigma$ is played along the history *h*. Player *i* considers all possible histories and evaluates them conservatively to foresee individual payoffs according to

4.3 $$\begin{aligned} x_i(\sigma ,s)=\min _{h\in {\mathcal {H}}(\sigma , s)}x_i(\sigma , h). \end{aligned}$$

Note the pessimism of the players. When uncertain about the subsequent development of the game, they assume that the remaining players will fabricate histories that are the least favourable to them. This is relevant both for *s* that are off the equilibrium path and for non-equilibrium $\sigma$ where subsequent deviations are possible.

While subgame perfectness can be formulated with these expectations, too, the resulting equilibria are different in general. Since the additional “information” comes from the past, the concepts of stationarity and stationary equilibria are not affected, the stationary equilibria remain the same and the recursive core equivalence result remains valid. Likewise, subgame consistency can be redefined in this environment, but we first clarify what is a relevant subgame.

### Equilibria

Once the game is defined we clarify what is meant under equilibrium behaviour in this game. We look at stationary subgame consistent equilibria (Kóczy 2015), a solution more inclusive than subgame-perfectness by ignoring subgames that are never reached, but one avoiding the folk-theorem like results of equilibria permitting nonstationary strategies. In the following we define these terms formally.

A subgame is trivial if it is identical to the game itself.

We rule out simultaneous deviations: For each deviation there exists $t'$ such that strategies for $t<t'$ are unchanged, and it is at $t'$ that some player *i* chooses a different action such as making of a different proposal (or none at all) at some time *t*, changing acceptance to rejection or vice versa, while the strategies can be quite different elsewhere.

#### Definition 3

(*Relevant subgame*) The original game is *relevant*. Given a strategy $\sigma$ and a current state *s*, a nontrivial subgame of a relevant subgame is *relevant* if it is played under $\sigma$ or after a profitable deviation $\sigma '$ by player *i* producing ${\mathcal {Q}}'$ and $\ell '$:

4.4 $$\begin{aligned} x_i(\sigma ,s)<x_i(\sigma ',({\mathcal {Q}}',\ell ',\varnothing ,\varnothing ,\varnothing )). \end{aligned}$$

Subgames that are irrelevant are never visited by rational players, as there are no strategy profiles that would justify entering them. For example, players, who can expect positive payoffs from the game will never enter a subgame where they would never exit.

Let $\sigma \vert _h$ denote the truncation of $\sigma$ to the subgame corresponding to *h*.

#### Definition 4

The strategy profile $\sigma ^*$ is a *subgame-consistent equilibrium* if for all relevant subgames $h^t$, $i\in N$, strategies $\sigma _i$ the corresponding restrictions $\sigma ^{*}\vert _{h^t}$ and $\sigma _i\vert _{h^t}$ to $h^t$ we have

4.5 $$\begin{aligned} x_i(\sigma ^{*}\vert _{h^t})\ge x_i(\sigma _i\vert _{h^t},\sigma ^{*}_{-i}\vert _{h^t}), \end{aligned}$$

that is, player *i*’s payoff cannot be higher by choosing another strategy $\sigma _i\vert _{h^t}$ in the subgame $h^t$.

Subgame perfect equilibrium strategies are also subgame-consistent.

#### Definition 5

A strategy $\sigma$ is *stationary* (or *Markov*) if it does not depend on time but only on the current state summarised by $h^t$. Formally: if for all $h_1,h_2$ and $t_1,t_2$ with $h_1^{t_1}=h_2^{t_2}$ we have $\sigma \vert _{h_1^{t_1}}=\sigma \vert _{h_2^{t_2}}$.

How can a strategy be Markov and depend on history at the same time? History, while describes a series of events, is actually the current interpretation and strategies depend on this current interpretation.

We study stationary subgame-consistent equilibria and therefore modify the subgame-consistency condition (Inequality 4.5) to include the stationarity property. In the formula we replace true history *h* by an arbitrary compatible alternative history *h*(*s*), with payoffs conditional on the current state *s* as given by Eq. (4.3).

4.6 $$\begin{aligned} x_i(\sigma ^{*}\vert _{h^t(s)},s)\ge x_i(\sigma _i\vert _{h^t(s)},\sigma ^{*}_{-i}\vert _{h^t(s)},s). \end{aligned}$$

The condition becomes clear now: it has implications not so much for the present, but for the reactions of the remaining players and it can be expressed conditional on the current state. Let $\sigma$ be a stationary strategy and $\sigma \vert _{s}$ its restriction to a state *s*.

A *stationary consistent equilibrium* $\sigma ^*$ is a strategy profile that is both subgame-consistent and stationary, that is, if for all relevant subgames corresponding to some *s* and for all *i* we have

4.7 $$\begin{aligned} x_i(\sigma ^{*}\vert _s,s)\ge x_i(\sigma _i\vert _s,\sigma ^{*}_{-i}\vert _s,s). \end{aligned}$$

### Results

Let $\Omega ^*(N,L,V)$ denote the set of outcomes resulting from playing stationary consistent equilibrium strategies.

#### Theorem 1

For a given network function form game (*N*, *L*, *V*) we have

4.8 $$\begin{aligned} C(N,L,V)\supseteq \Omega ^*(N,L,V). \end{aligned}$$

The proof is by induction using a number of auxiliary results.

#### Lemma 2

Assume that $\omega \in \Omega ^*(N,L,V)$. Then there exists a stationary-consistent equilibrium $\sigma _0$, where $\omega$ is formed without delay.

#### Proof

Since $\omega \in \Omega ^*(N,L,V)$, there exists a stationary-consistent strategy $\sigma$ that produces $\omega$. Now consider a modification $\sigma _0$, where the outcome $\omega$ is proposed at time 0 and where such a proposal is accepted by all players. Otherwise $\sigma$ and $\sigma _0$ coincide. Then we claim that $\sigma _0$ is a stationary-consistent strategy.

To see this, consider any deviation from $\sigma _0$ resulting in a state *s*. At *s* the two strategies coincide and therefore the outcome of the game will be the same. If the deviation is profitable under $\sigma _0$, it must be profitable under $\sigma$, too. Contradiction. Therefore $\omega \in \Omega ^*(N,L,V)$ may form without delay. $\square$

#### Lemma 3

Assume that Theorem 1 holds for all network function form games with up to $k-1$ players. Then for all games with $\left| N\right| =k$

4.9 $$\begin{aligned} C(N,L,V)\supseteq \Omega ^*(N,L,V). \end{aligned}$$

#### Proof

Either $\Omega ^*(N,V)=\varnothing$ and the result is trivial, or there exists a stationary consistent equilibrium strategy profile $\sigma$ producing $\omega (\sigma ,h)=(x(\sigma ,h),{\mathcal {P}}(\sigma ,h),\ell (\sigma ,h))$ such that $\omega (\sigma ,h)\in \Omega ^*(N,L,V)$ for some sequence of possible histories $h\in {\mathcal {H}}(\sigma ,\varnothing )$. We assume that $\omega (\sigma ,h)\not \in C(N,L,V)$ and prove contradiction.

If $\omega (\sigma ,h)\not \in C(N,L,V)$ then there exists a profitable deviation ${\mathcal {D}}, \ell$ by some set *D* of players in the cooperative game. First look at the residual game of this deviation. If the core is empty, the Deviating players expect the worst possible residual outcome and even assuming this, the deviation is profitable. This implies that in a stationary consistent equilibrium the corresponding deviation is profitable, too.

Now consider the case of a nonempty residual core $C({\overline{D}},L^{\ell }_{{\overline{D}}},V_{{\overline{D}}}^{\ell ,{\mathcal {D}}})$. The residual game $({\overline{D}},L^{\ell }_{{\overline{D}}},V_{{\overline{D}}}^{\ell ,{\mathcal {D}}})$ has fewer players and therefore we can use the inductive assumption to conclude that $C({\overline{D}},L^{\ell }_{{\overline{D}}},V_{{\overline{D}}}^{\ell ,{\mathcal {D}}})\supseteq \Omega ^*({\overline{D}},L^{\ell }_{{\overline{D}}},V_{{\overline{D}}}^{\ell ,{\mathcal {D}}})$.

According to the definition of the recursive core, players in *D* only deviate if this deviation is profitable for each outcome in $C({\overline{D}},L^{\ell }_{{\overline{D}}},V_{{\overline{D}}}^{\ell ,{\mathcal {D}}})$. Since $\Omega ^*({\overline{D}},L^{\ell }_{{\overline{D}}},V_{{\overline{D}}}^{\ell ,{\mathcal {D}}})$ is a subset, the same deviation is profitable with respect to $\sigma$ for any subgame-consistent strategy that may be applied in the subgame that entails. So we get the desired contradiction; in other words $\sigma$ cannot be subgame-consistent. $\square$

#### Proof of Theorem 1

The proof is by induction. The result holds for trivial, single-player games. Assuming that the result holds for all $k-1$ player games, the result for *k*-player games Lemma 3 gives the desired induction step. $\square$

## Applications

Our model is driven by externalities over the network: the idea that the formation of a link has effects well beyond the nodes or players it connects—not to be confused with network externalities or network effects describing the phenomenon that the value of a product depends on the number of people owning it.

Externalities are often characterised by the sign of the external effects. As such, we may talk about positive externalities when the formation of a link is generally of positive value to others, or about negative externalities when the new link harms others. We will illustrate both cases with a simple story. In the first, second-degree neighbours are of importance and so friendships our friends make are beneficial to us, too. In the second, connections our contacts make increase the risk of an infection, thereby harming us.

### Favour network

We consider an even simpler example of a network, where link formation creates externalities and a wider cooperation can result in more efficient outcomes.

We take the example of a favour network consisting of individuals who maintain friendships at some cost *c*. For simplicity we assume that a friendship is mutual, but the costs of maintaining the friendship are not necessarily shared equally: in the usual TU fashion we envisage a complex system of transfers of who buys which drink to maintain the network. Having many friends is great, but now we are interested in friends’ friends. When a friend’s friend is hiring and we want to apply for the job, the friend can put in a good word for us. The same would not work if we would make direct contact as praising ourselves is not so credible. Similarly, more distant relations may have too little information about us. In sum, the benefit of the network is the number of secondary friends a player has. Examples of such a network include the referral network studied by Stupnytska and Zaharieva (2017). In the following we formalise this rule, define the payoff function and determine the emerging equilibrium networks.

Let $N_i=\left\{ j\vert j\in N, \exists {\overline{ij}}\in \ell \right\}$ denote the neighbours of node *i* and let $N_i^2=\bigcup _{j\in N_i}N_j{\setminus }\left\{ i\right\}$ denote the secondary connections of *i*. Let $d_i=\left| N_i\right|$ denote the degree of node *i*. Similarly, let $d^2_i=\left| N^2_i\right|$ denote the secondary degree of node *i*. Note that the payoff does not depend on the coalition structure. The payoff of coalition *C* embedded in partition ${\mathcal {P}}$ given the network $\ell$ is

5.1 $$\begin{aligned} V_C(\ell )=\sum _{i\in C}\left( d^2_i-\frac{d_ic}{2}\right) . \end{aligned}$$

Note the absence of outside nodes.

We would like to find the core of this game.

#### Proposition 4

The core of the favour network game may only contain efficient networks.

#### Proof

Since the payoff function does not depend on the partition of the players, the game is cohesive, that is, the grand coalition can achieve any configuration. If the network is not efficient, a deviation by the grand coalition can strictly improve it and can strictly increase the payoff of each of the players. $\square$

In the following we determine the efficient network. We discuss three main cases:

*Tree* If the underlying network $\ell$ is a tree we show that it must be a star. Assume that this is not the case and that $i\in \arg \max _j d_j$. Moreover let *k* be a leaf not connected to *i*, but to *j*. Since *k* is a leaf, $d_k^2=d_j-1$. Now modify $\ell$ such that the link between *j* and *k* is moved to *i* and *k*. Since *k* is still a leaf we get $d_k^{'2}=d'_i-1=d_i>d_j-1=d_k^2$, where the $d'$-s refer to values in the modified network. Since only these and the reciprocal indirect connections are affected, the net gain is positive. Therefore all nodes must be connected to the node with the highest degree resulting in a star.

**Fig. 11:**
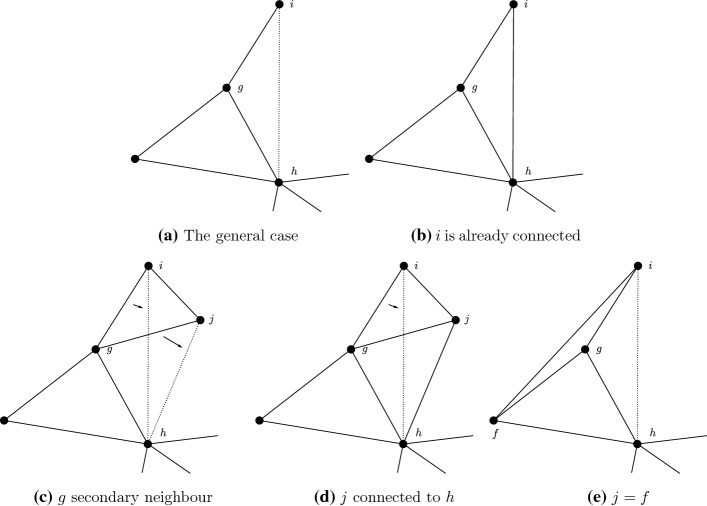
Improvements in graphs with triangles

*Graph with triangles* Now consider the case when the network is not a tree. Firstly assume that the underlying graph $\ell$ contains triangles. Consider a triangle $T=\left\{ f,g,h\right\}$, such that, without loss of generality $h\in \arg \max _{i\in T}d_i$. We will show that the value of the network increases if we move the links (except from those from *f* and *h*) pointing to *g* to *h* instead. To be more precise: if there are such *i* that are not connected to *h* then the value of the network can be increased. We discuss 5 cases (Fig. 11).

*Case 1: No complications* Consider a node *i* such that ${\overline{ig}}\in \ell$. Moving this link from *g* to *h* the number of secondary contacts in *T* remain the same: previously *f* and *h*, now *f* and *g*. On the other hand if we move the similar links for all $i\in N_g{\setminus } T$, the former secondary contacts via *g* remain secondary contacts via *h*. Those in $N_h{\setminus } T$ are new secondary contacts, while the number of links has not increased. If we have players outside T, the gain is strictly positive.

*Case 2: Already connected* If some of these *i* nodes are already connected to *h* there is no benefit to moving the links to *h*: while double links are permitted by our formalism, in this example they bring no benefits. In this case no links are shifted. If all such *i* nodes are connected to *h* then the value cannot be increased, at least this way.

*Case 3: g is already a secondary neighbour* When *g* is already a secondary neighbour moving the link to *h* loses *h* as a secondary neighbour, but to no gain, as *g* is already in $N^2_i$. How is this possible? There exists *j* with ${\overline{ij}}, {\overline{gj}}\in \ell$. But then following case 1 we move both links: as a result we do lose both *g* and *h* as secondary links, but get them back both. At the same time the benefits of $N_h{\setminus } T$ as new connections still apply.

*Case 4: j is already connected to h* If *j* is already connected to *h* we cannot move both links, but, like in Case 2, the link is already there and so, if we wish, the roles of the links ${\overline{gj}}$ and ${\overline{hj}}$ can be switched.

*Case 5:* $j=f$ It is perhaps useful to specially mention the case when $j=f$. Actually, this case is no different from the rest. Of course, *f* is connected to both *g* and *h*, so we really have a special case of Case 4.

In a similar fashion we can move links to *f* to *h*, too. As a result triangles are connected to the rest of the network via one of their vertices only.

*Larger cycles* Now we show that larger cycles cannot be part of an efficient network. For the moment assume that there are larger cycles, too. Due to the previous result, the cycle may only share vertices and not arcs with triangles. Consider the smallest cycle of length at least 4, *C*—this has at least four nodes: let *h*, *i*, *j* and *k* nodes following each other on the cycle and let $h\in \arg \max _{m\in C} d_m$ be one of the points with the highest degree in the cycle. By the result that ${\overline{hi}}$ is not part of a triangle, $N_h$ and $N_i$ are disjoint. Then consider the following modification to the network: move the arc linking *j* and *k* to link *j* and *h*. After the change *j* has $d_h+d_i$ secondary neighbours, while before the change at most^1^$d_k+d_i<d_h+d_i$. Therefore if the graph has larger cycles, it can be made more efficient by creating a triangle and thereby breaking the cycle. A repetition of this step eliminates all cycles of length 4 or more.

After the elimination of large cycles, and following the recommended improvements, we get a graph, which looks a bit like a tree, but with some triangles attached to some vertices. Thanks to this similarity, we can improve this graph similarly to the improvement applied for trees:

Select $i\in \arg \max _j d_j$. With more than 2 players and a connected graph we either do not have triangles or $d_i>2$ in which case *i* cannot be one of the non-connecting vertices (the *f*’s and the *g*’s) of a triangle. Let *f* and *g* such non-connecting vertices of a triangle $T=\left\{ f,g,h\right\}$. Now modify the graph so that ${\overline{fh}}$ and ${\overline{gh}}$ are moved to ${\overline{fi}}$, ${\overline{gi}}$. As before, by moving to a node with a higher degree, both *f* and *g* have more secondary connections. While the direct connections $N_h{\setminus }\left\{ f,g\right\}$ of *h* lose them, those in $N_i$ gain them and by assumption $d_i\ge d_h$.

Once we are done with the triangles, we have a node with many triangles attached to it, but for the rest, the graph is just like a tree. So let *k* be a leaf not connected to *i*, but to *j*. Since *k* is a leaf, $d_k^2=d_j-1$. Now modify $\ell$ such that the link between *j* and *k* is moved to *i* and *k*. Since *i* is still a leaf we get $d_k^{'2}=d'_i-1=d_i>d_j-1=d_k^2$, where the $d'$-s refer to values in the modified network. Since no other indirect connections are affected, the net gain is positive. Therefore all nodes must be connected to the node with the highest degree.

**Fig. 12:**
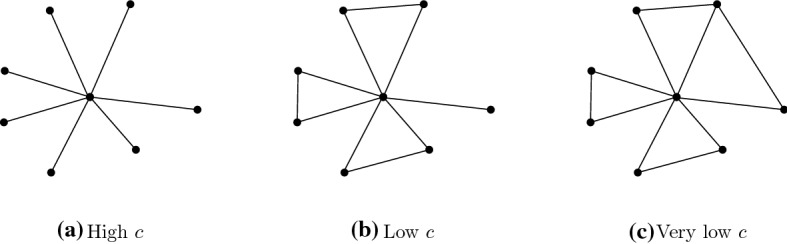
Efficient networks

So far we have only looked at improvements that did not affect the number of direct connections, we merely rearranged them to have a more efficient structure. As a result we have a player at the centre and all other $n-1$ players are linked to it. Some of these outer players *f*, *g* are directly connected. Such connections are never needed to have each other as secondary connections as this works via the central player. Outer links are used to have the central player as a secondary connection. The added value of such a link is therefore $4-c$ if neither *f* nor *g* is connected to other non-central players, the value is $2-c$ if one of them is connected and $-c$ if both. For high *c* these links are severed, for low values of *c* non-central players link up in pairs, and if *n* is even (so that $n-1$ is odd) the remaining non-central player links to another only if $c<2$. The links to the centre only break if $c>2(n-2)$ (Fig. 12).

Therefore if $c>2(n-2)$ we get an empty network, for $2(n-2)> c> 4$ we get a star, for $4> c$ we get a flower: if *n* is even, for $c>2$ it has a stem, otherwise a double petal.

#### Proposition 5

The core of the favour network game is empty.

#### Proof

The next question is stability. Consider a deviation by a single player forming a singleton coalition. If this player forms or keeps no links, it has a zero payoff. Let us see if it can have a higher payoff. Suppose it keeps a link with its highest-degree neighbour. Since the residual game will be similar to the original one, the players form a star or a flower. If so, it is always better to form it “around” the player with the external link. Thereby the deviating player becomes a peripheral player in a star with a payoff $n-2-\frac{c}{2}$. The total value of a star is $(n-1)(n-2)-(n-1)c$. Since the star is formed by *n* players, there is a player with a payoff of at most $\frac{(n-1)(n-2)-(n-1)c}{n}$, therefore the deviation is profitable if

$$\begin{aligned} n-2-\frac{c}{2}>\frac{(n-1)(n-2)-(n-1)c}{n} \end{aligned}$$

This is satisfied when $c>2(n-2)$, but we have not tested the stability of the residual core. If it empty, the deviating player must expect the worst of all possible reactions, including the one where links to it are broken and therefore his payoff is 0. To check this, consider a more general case with *k* players deviating. It is easy to see that these players will all be peripheral players who do not want to change the underlying network, only the distribution of the payoffs, so that all these players will keep their links to the central player and then efficient and therefore only possible reaction in the residual game is a star around that player. The question is: will this player keep the links to the deviated players. What causes the problems? While the total value of the network does not change, the central player, by maintaining the external links, subsidizes the deviated players more and more. As the number of departed players increases the residual players’ benefit per link to the deviating players decreases, while the associated costs remain the same. The links remain profitable only if

$$\begin{aligned} n-k-1>\frac{c}{2} \end{aligned}$$

where $0<k<n$. For some *k* this will be violated and then the deviations are not profitable any more. Consider a deviation by $k-1$ peripheral players: the residual core is nonempty and the deviation will be profitable. Therefore the recursive core of this game is *empty*. $\square$

Note that this finding is driven by the fact that the central player must sacrifice himself to the benefit of others: Normally others compensate him for this, but selfish players may deviate and stop such transfers. In reality such a central player has a very strong position and gets rewarded for the favours he can provide. In the following example we make these rewards explicit by assuming that, upon forming a link between players *i* and *j*, player *i* must pay a transfer to *j* that is proportional to $d_j-1$. As a result, a central player gets a high transfer, while a leaf gets nothing. Then the payoff of coalition *C* embedded in partition ${\mathcal {P}}$ given the network $\ell$ is

5.2 $$\begin{aligned} V_C(\ell )=\sum _{i\in C}\left( d^2_i-\frac{d_ic}{2}+\left( d_i(d_i-1)-\sum _{j\in N_i}(d_j-1)\right) t\right) , \end{aligned}$$

where $t<1$ is the compulsory transfer for using an intermediary.

#### Proposition 6

The core of the modified favour game is not empty if *t* is sufficiently high $t>\frac{c+2}{2n}$.

#### Proof

Firstly observe that the modification merely introduces transfers among players, so that the value of the grand coalition does not change. In particular, the efficient structures remain the same. We may therefore focus on the issue of stability. We limit our attention to star structures; the case when $c<4$ is similar.

Consider a star, and consider a deviation by *k* peripheral players. What happens in the residual game? The former central player has already *k* connections to the deviating players. Due to our assumptions that no new links may form between coalitions, no other player can have external links. By linking to this player the remaining $n-k-1$ players do not only get a very high payoff, but they also increase the value of this central player’s services to the deviating players. Formerly this was positive externality they could not benefit from, but now the deviators must pay a fee for it. So if the residual core is not empty, it keeps the pre-deviation structure. Is this core non-empty? To see this, first compare the payoffs of players in different positions (without the possible transfers within the coalition). We will show that the central player earns more. To see this, observe the following: What a player earns only depends on the network. The network has not changed due to the deviations. At last: the network, and the payoffs (recall we ignore transfers) are symmetric among the peripheral players. Therefore if we show that the average payoff is higher than the peripheral players’ payoff this shows the result.

A player on the periphery has a value $n-2-(n-2)t-\frac{c}{2}$, while an average player has $\frac{(n-1)(n-2)-(n-1)c}{n}$. We want to show

5.3 $$\begin{aligned} n-2-(n-2)t-\frac{c}{2}< & {} \frac{(n-1)(n-2)-(n-1)c}{n} \end{aligned}$$

5.4 $$\begin{aligned} \frac{c+2}{2n}< & {} t \end{aligned}$$

That is, if *t* is sufficiently large, the central player earns more. In such a case the central player has no incentives to deviate and become a peripheral player, while a player can only become central by cooperation with all other players. This holds both in the original game and in the residual game, since the underlying networks are the same.

For lower *c* values we must also check the incentives to keep peripheral links but these will be kept for the very same reason they were created in the efficient network. $\square$

### Contagion network with social preferences

Our next application is motivated by the recent Covid-19 epidemic and is a clear example of negative externalities. While various mathematical models have been introduced to study the optimal response to an epidemic (Parvin et al. 2012; Sharomi and Malik 2017), our model takes the citizens’ perspective. The network models social contacts of individuals, where the tradeoff is between the benefit of having friends and the risk of getting infected. It is assumed that an individual may get infected by an involuntary contact and may spread the infection to others in his or her social network.

This problem is rather different from other instances of bad networks. It is not really related to the literature on dark or covert networks (Milward and Raab 2006; Husslage et al. 2013), where the nodes of the bad network are aware of the fact that their contacts are bad and are contributing to the maintenance of this network. In our model the network is a positive message but carries the risk of spreading the disease. Even if only a small fraction of the population is infected, network results, known as the small world phenomenon suggest that the social distance from infected people may be smaller than what one would think (Vieira et al. 2010). Put it differently: nodes do not know if they or their neighbours are infected and the threat is near.

We use the notation of the previous subsection by letting $d_i$ denote the degree of node *i*. In addition we use $d^{\infty }_i$ to denote the number of nodes connected to *i*: in other words the size of *i*’s component.

For a coalition *C* the payoff is

5.5 $$\begin{aligned} V_C(\ell )=\sum _{i\in C}\left( s_i(d_i)-d^{\infty }_i\right) , \end{aligned}$$

where $s_i$ is a concave, weakly increasing function that we call player *i*’s *sociability function*. The sociability function expresses the benefit from being social, keeping in touch with people. We assume that the function is increasing (the more, the merrier). At this point we are also very pragmatic and assume that it is only the number of contacts that matters.

Expression $V_C(\ell )$ models the payoff from a long-term strategy of keeping in touch with a chosen set of other players (and avoiding contact with everyone else). At the same time, people who contact the epidemic via involuntary encounters will eventually spread the infection to everyone in the same network component.

It is clear from looking at an individual’s payoff (coalition $\left\{ i\right\}$) that a typical player is either extremely social, wanting to keep in touch with everyone or has a bound on the optimal number of connections where the additional risk exceeds the benefit of seeing one more person. We will ignore extremely social people or just assume that their bound is the number of players.

In the following we present some simple results regarding this problem. The first result is almost trivial.

#### Lemma 7

An core outcome has a network with fully connected components.

#### Proof

It is clear that each player benefits from having more contacts while keeping the risk of infection at the same level. The network with fully connected components can actually be obtained by a deviation by *N* partitioned into coalitions corresponding to the components. The coalitions will continue to have no external connections but will build all internal links. No player is harmed by the change so the deviation is profitable. $\square$

#### Homogeneous players

First we look at the case where $s_i=s_j$ for all $i,j\in N$. In this case all players have the same bliss point. Let *k* denote this number of ideal neighbours, where the marginal benefit of an additional neighbour is less than the cost of additional risk.

Consider a deviation by some coalition *S* forming partition $\mathcal {S}$. Then each coalition $C\in \mathcal {S}$ with $\left| C\right| >k$ will severe all external links. To see this, we construct a network that gives a higher payoff for the coalition. If there is a player *i* with more than *k* neighbours including *x* outside *C*, then severing ${\overline{ix}}$ increases the coalitional payoff. Now consider player *j* with less than *k* neighbours, including *y* in $N{\setminus } C$. Since *j* has less than *k* neighbours, there is also an $h\in C$, such that ${\overline{jh}}\not \in \ell$. Then the network, where ${\overline{jy}}$ is severed and ${\overline{jh}}$ is created gives a higher payoff: the number of *j*’s neighbours does not change, $h's$ increase, while $n^{\infty }$ stays or decreases for all in *C*. On the other hand it may be that $y\in C'\subset S$ is harmed. Notice, however that as soon as *C* breaks all external links, it does not experience externalities from other coalitions. In other words, if $\mathcal {S}$ can deviate profitably, then a profitable deviation for $\left\{ C\right\}$ exists, where it separates itself from all other players.

When *k* is the ideal number of neighbours, a player has the highest payoff in a component with $k+1$ players, where he is connected to all other members. In the symmetric case the same applies to all other members of this component. Therefore we have the following observation.

##### Remark 1

The per-member payoff is the highest in a coalition corresponding to a component with exactly $k+1$ players.

##### Corollary 8

A deviation by a coalition of size $k+1$ forming a fully connected component is profitable if at least one of the members belongs to a coalition of a different size.

##### Theorem 9

The core consists of a single outcome with the players partitioned into fully connected components of size $k+1$ and each player getting the same payoff.

##### Corollary 10

The core is nonempty if and only if $\left| N\right|$ is divisible by $k+1$.

#### Heterogeneous players

Just as in the homogeneous case, each player *i* has an optimal number of contacts $k_i$. The following result is fairly trivial.

##### Lemma 11

If there exists a partition ${\mathcal {P}}$ of *N* such that each coalition $S\in {\mathcal {P}}$ contains only players with optimal number $\left| S\right| -1$ then the outcome where the network consists of fully connected components according to ${\mathcal {P}}$ and each player getting his own payoff is the only element of the core.

These conditions are rather special, it is generally not possible to group players into such components. Consider the following, very simple example.

##### Example 4

Consider a network with only 2 players, 1 and 2. Without loss of generality $s_1(0)=s_2(0)=2.8$, but $s_1(1)=3$ and $s_2(1)=4$. Clearly, Player 1 prefers to be solitary as the additional risk is higher than the marginal benefit of keeping in touch with a friend. Therefore $k_1=0$. On the other hand, 2 is happy to be in touch with another player. Two networks are feasible corresponding to two singletons ($\ell _1$) or a pair ($\ell _2$). Then $v(\left\{ 1\right\} ,\ell _1)=2.8$, $v(\left\{ 2\right\} ,\ell _2)=2.8$, while $v(N,\ell _2)=3+4-2=5$, therefore the core network is segregated.

On the other hand, if the payoffs are somewhat different, $s_1(0)=s_2(0)=2.8$, but $s_1(1)=4$ and $s_2(1)=5$, the payoffs of the singletons remain the same, but the pair gets $v(N,\ell _2)=4+5-2=7$, therefore the more social player can compensate the other and form a component of her ideal size.

It is good to see that a nonempty core does not require the extremely rare structure required in Lemma 11: so an equilibrium network may contain components containing heterogenous players. Do those components at least contain similar players? In some sense, probably yes, but their preferred component size is a poor indicator for that. A player with a very small optimal size may have nearly the same benefit for every additional contact, while another characterised by a larger *k* may get practically no benefit from additional contacts. The second has a higher *k* value, but is very difficult to compensate for the larger component, while the first prefers larger components when these come with a little transfer.

Let $$\Delta s_i(m)=s_i(m)-s_i(m-1)$$.

##### Theorem 12

Consider a player set *N* such that $\Delta s_1(m)\le \Delta s_2(m)\le \dots \le \Delta s_{\left| N\right| }(m)$ for all *m*. Then if the core is not empty then there exists a core outcome with a network that sorts players into components according to their sociability function *s*.

##### Proof

There is a natural ordering of coalitions according to size. Players with a low index prefer to be in small coalitions, while those with a large index, in large coalitions. Now assume that the theorem is false. This means that some social players ended up in small, and more introvert players in large coalitions. In particular, there are two players, *i* and *j* in two neighbouring coalitions ${\mathcal {P}}(i)$ and ${\mathcal {P}}(j)$ of different sizes ($d_i+1> d_j+1$), such that they are “mixed up”: for example $s(i)> s(j)$, while $d_i+1=\left| {\mathcal {P}}(i)\right| \le \left| {\mathcal {P}}(j)\right| =d_j+1$. Take this pair and consider a deviation with the components as coalitions but two players exchanged. In this deviation, the degrees of other players do not change, so we can focus on *i* and *j*, where $d'_i=d_j$ and $d'_j=d_i$. Then $V_{{\mathcal {P}}(i)\cup {\mathcal {P}}(j)}(\ell ')-V_{{\mathcal {P}}(i)}(\ell )-V_{{\mathcal {P}}(j)}(\ell ) =s_i(d'_i)-d_i^{'\infty }+s_j(d'_j)-d_i^{'\infty }-(s_i(d_i)-d_i^{\infty }+s_j(d_j)-d_i^{\infty }) =s_i(d_j)+s_j(d_i)-s_i(d_i)-s_j(d_j) =(s_i(d_j)-s_i(d_i))-(s_j(d_j)-s_j(d_i)) =\sum _{m=d_i+1}^{d_j}\left( \Delta s_i(m)-\Delta s_j(m)\right) >0$, where the last inequality follows from our assumption and that $s(i)> s(j)$. $\square$

When there are multiple components of the same size, it does not matter how the players are distributed resulting in additional—payoff-equivalent—core outcomes.

## Remarks

We have introduced a rather general model to allow for coalitional improvements in a network with possible transfers among coalition members. We have used two very simple games, a favour network and a social network with contagion to illustrate this model.

In the first version there was a tradeoff between direct costs and indirect benefits, while in a second model players with many friends got transfers in exchange of their services as intermediaries. While such transfers are quite natural even in the settings of such personal connections, our model fits rather well other situations. Consider the hub-and-spoke network of air travel. There are natural differences: direct connections are the most valuable, so let *c* denote the difference between the cost and the benefit of the connections. The problem is not very interesting if the benefits exceed the costs—we will have a complete network. On the other hand we see an increasing cost of flights to major hubs. While technically it is not airports paying transfers to others, minor airports heavily subsidise flights to major destinations as it makes them attractive to travellers (Borenstein 1989). Do we see our model confirmed in real life? Yes, in the sense that our equilibrium network is a hub and spoke network, but our example did not allow for heterogeneity among the players or the emergence of transportation hubs (Konishi 2000). In real life popular hubs do not simply charge more, but due to congestion adding new flights might become prohibitively costly or even impossible due to capacity constraints, at lest in the short run. We are still working on the problem with explicit capacity constraints, but it is clear that the network will be less simple.

In our model players can, at a given, exogenous cost build a connection for sure. Brueckner (2006) considers a friendship network where players choose the effort level and the formation of a link is probabilistic, the higher the effort (and the associated cost) the higher the probability that the link forms. While our model is based on the assumption that coalitions have certain powers in building/severing links, the joint optimisation of connections may still work in such a probabilistic setting.

Our model of the social network with the risk of contagion is based on the experience and restrictions on social contacts employed in many countries in relation to the Covid-19 epidemic that started in the end of 2019. Due to the relatively long latency period before the infection one could get infected by meeting a seemingly perfectly healthy person. As a precaution measure, avoiding social contacts was recommended and various formal restrictions have been introduced in various countries. These included complete curfew, where one could only contact members of the household (by law) to less its less formal versions. Our approach is motivated by New Zeeland’s social bubble approach that allows people to interact with a small group of family and friends while in lockdown. During the high alert phases of the epidemic the bubbles have been restricted to single households, but by mid April 2020, as the threat of infection diminished, citizens were allowed to expand their bubbles, “keep it exclusive, though and keep it small” (Prime Minister Jacinda Arden on 16 April)—very much in line with the core networks balancing the need to socialise with the infection risk.

These examples have been chosen for their simplicity. There are also numerous other possible applications for this model. The balancing groups in electric power networks (Csercsik and Kóczy 2017) rarely change but such changes could also bring in new power line constructions. For the natural gas pipeline network, the construction of the Nord Stream 2 pipieline directly connecting Russia and Germany (Sziklai et al. 2018) is a prime example where a consortium backed by a coalition of countries constructs a pipeline—followed by a reorganisation of the complementary coalition including the construction of the Trans-Anatolian and Trans-Adriatic Pipelines (TANAP and TAP). Sziklai et al. (2018) also argue that the closure of an existing external connection, the Ukrainian corridor may follow the construction.

We may also think of road infrastructure planning where regions act as players (Wang and Zhang 2017). Or a residential neighbourhood with a trade-off between being accessible but not too central where stopping through traffic by closing the central area may divert traffic to parallel routes who may respond by similar measures. As a final example, the selling of a subsidiary does not only mean that the internal processes are reorganised, but the entire supply chain must and does react to the new market conditions.

## Footnotes

1 Note that *i* and *k* may have common neighbours.
